# Intracluster correlation coefficients for sample size calculations related to cardiovascular disease prevention and management in primary care practices

**DOI:** 10.1186/s13104-015-1042-y

**Published:** 2015-03-20

**Authors:** Jatinderpreet Singh, Clare Liddy, William Hogg, Monica Taljaard

**Affiliations:** Bruyère Research Institute, C.T. Lamont Primary Health Care Research Centre, 43 Bruyere St (Annex E), Ottawa, ON K1R 7G5 Canada; Department of Family Medicine, University of Ottawa, 43 Bruyere St, Ottawa, ON K1N 5C8 Canada; Clinical Epidemiology Program, Ottawa Hospital Research Institute, 1053 Carling Ave., Ottawa, ON K1Y 4E9 Canada; Department of Epidemiology and Community Medicine, University of Ottawa, 451 Smyth Road, Ottawa, ON K1H 8M5 Canada

**Keywords:** Intracluster correlation coefficient (ICC), Cardiovascular disease, Primary care

## Abstract

**Background:**

Few studies have comprehensively reported intracluster correlation coefficient (ICC) estimates for outcomes collected in primary care settings. Using data from a large primary care study, we aimed to: a) report ICCs for process-of-care and clinical outcome measures related to cardiovascular disease management and prevention, and b) investigate the impact of practice structure and rurality on ICC estimates.

**Methods:**

We used baseline data from the Improved Delivery of Cardiovascular Care (IDOCC) trial to estimate ICC values. Data on 5,140 patients from 84 primary care practices across Eastern Ontario, Canada were collected through chart abstraction. ICC estimates were calculated using an ANOVA approach and were calculated for all patients and separately for patient subgroups defined by condition (i.e., coronary artery disease, diabetes, chronic kidney disease, hypertension, dyslipidemia, and smoking). We compared ICC estimates between practices in which data were collected from a single physician versus those that had multiple participating physicians and between urban versus rural practices.

**Results:**

ICC estimates ranged from 0 to 0.173, with a median of 0.056. The median ICC estimate for dichotomous process outcomes (0.088) was higher than that for continuous clinical outcomes (0.035). ICC estimates calculated for single physician practices were higher than those for practices with multiple physicians for both process (average 3.9-times higher) and clinical measures (average 1.9-times higher). Urban practices tended to have higher process-of-care ICC estimates than rural practices, particularly for measuring lipid profiles and estimated glomerular filtration rates.

**Conclusion:**

To our knowledge, this is the most comprehensive summary of cardiovascular-related ICCs to be reported from Canadian primary care practices. Differences in ICC estimates based on practice structure and location highlight the importance of understanding the context in which external ICC estimates were determined prior to their use in sample size calculations. Failure to choose appropriate ICC estimates can have substantial implications for the design of a cluster randomized trial.

## Background

Cluster randomized trials are increasingly being used in primary health care research [1]. In a cluster randomized trial, groups of individuals (e.g., primary care practices, hospitals, communities), rather than individual patients themselves, are randomly allocated to either an experimental or control intervention. Cluster randomization is required when interventions are necessarily delivered at the group or cluster level, such as an initiative that introduces specialist nurses into primary care practices. In cases where an intervention is delivered on an individual level, cluster randomization may be preferable due to logistical, practical, or scientific reasons [2].

It has become well known that cluster randomized trials are statistically less efficient than trials using individual randomization, as individual responses within a cluster are usually positively correlated [2,3]. The degree of correlation is usually measured by the Intracluster Correlation Coefficient (ICC). To account for intracluster correlation, sample sizes required for cluster randomized trials must be increased to reach the desired power [2]. Sample size calculation formulas for cluster randomized trials are widely available [2,4-6].

One method to account for clustering in sample size estimates, assuming constant cluster sizes, involves using the variance inflation factor (VIF) [2]. The VIF for a cluster randomized trial with a simple parallel design is a function of the cluster size (m) and the intracluster correlation coefficient (ICC) which is denoted by ρ: VIF = 1 + (m ‐ 1)ρ. The ICC represents the proportion of variance in a given outcome that can be explained by the variation between clusters, and is given by ρ = σb2/(σb2 + σw2), where σb2 is the between cluster variance and σw2 is the within cluster variance. An alternative expression for the VIF when the cluster sizes vary is provided by Donner, Birkett and Buck [7].

To conduct sample size calculations for a planned cluster randomized trial, advance estimates of the ICC are required. Estimates are often made available from previously published reports of cluster randomized trials evaluating similar outcomes. Despite recommendations to report ICC values in published reports of cluster randomized trials, [8] it is still challenging to find applicable ICC values to aid in the design of future trials [9].

One concern in using external ICC estimates is whether they are appropriate for the planned cluster randomized trial. If an inaccurate estimate for the ICC is used, the resulting sample size estimate may be either too large or too small. Several studies have analyzed determinants of ICCs [9-12]. Choosing appropriate estimates for ICCs is of particular concern in primary care research, where practice characteristics often vary widely across various domains, including rurality, physician remuneration model, and practice structure (i.e., solo or multiple physician practices).

We recently conducted a large primary care quality improvement initiative in 84 primary care practices across Eastern Ontario, Canada [13]. Through this initiative, we have collected data from 5,140 patients who either have, or are at high risk of developing, cardiovascular disease. The main objective of this paper is to use this rich dataset to: a) report ICC values for a range of process of care and clinical outcome measures related to cardiovascular disease management and prevention, and b) investigate differences in ICC values based on: i) number of physicians per practice (i.e., solo versus multiple physician practices) and ii) urban versus rural practices. Understanding the potential impact that these practice characteristics have on ICC values is important, as a failure to take such factors into account can lead to studies that are inadequately powered.

## Methods

### Improved delivery of cardiovascular care (IDOCC) through outreach facilitation trial

The Improved Delivery of Cardiovascular Care (IDOCC) through Outreach Facilitation trial was designed as a stepped wedge cluster randomized control trial to support 84 primary care practices in improving their delivery of evidence-based cardiovascular care for patients at high risk [13]. IDOCC used trained facilitators who worked with practices for 12–24 months to incorporate elements of the chronic care model into daily practice routines to improve secondary preventive care for heart disease, stroke, peripheral vascular disease, chronic kidney disease, and diabetes. Primary care practices were located throughout the Champlain Local Health Integration Network (Ottawa and its surrounding communities) of Ontario, Canada, a culturally diverse region with a population of 1.2 million people who have chronic disease burdens and patient health outcomes that are comparable to Ontario and the rest of Canada. Canada has a publicly funded universal health insurance system, which is often referred to as “Medicare”. Detailed information about the recruitment, participants, and data collection can be found elsewhere [13].

In brief, all practices within the Champlain Local Health Integration Network were invited to participate in IDOCC. The Champlain Local Health Integration Network was systematically divided into nine geographic regions, which were grouped together into strata by their location (i.e., west, central, and east). A computer generated randomization approach was used to assign each region within each stratum into one of the three steps of the stepped wedge design. Practices were enrolled in the trial if at least one physician from the practice agreed to participate. In total, 194 physicians in 93 practices were enlisted to participate, with nine practices dropping out prior to the initiation of the study.

Data were abstracted by chart auditors from a random sample of patient medical charts from participating practices, in order to assess each practice’s adherence to evidence-based guidelines for CVD care. Eligible patients for the chart audit were those over 40 years of age who met at least one of the following criteria: (1) CVD including coronary artery disease, cerebrovascular disease (documented stroke and/or transient ischemic attack), or peripheral vascular disease; (2) diabetes mellitus; (3) chronic kidney disease; and/or (4) be at high risk for CVD based on the presence of at least three of the following cardiovascular risk factors: age (males ≥ 45, females ≥ 55), smoker, hypertension, and dyslipidemia. ICC estimates presented in this paper were calculated from baseline data collected from 5,140 eligible patients (average cluster size of 61 patients/practice; range: 18 to 66). The mean cluster sizes were similar across the 9 regions, ranging from 60 to 65. In the IDOCC study, patient identifiers were available to uniquely link patients to specific practices, but not to specific physicians within practices. In group practices, it is not uncommon for a patient to be seen by multiple physicians in cases where their primary family physician is unavailable. Also, many group practices have a nurse on staff that performs certain clinical measures (e.g., blood pressure, waistline, weight, etc.) for all patients that are treated in a given practice. As such, inferences were to be made with respect to the practice, rather than individual physicians. For the purpose of presenting ICC estimates, we therefore calculated ICCs within practices. A similar approach was used in several other studies which presented ICC estimates in primary care settings [9,14,15].

Chart auditors collected data related to recommendations from the Champlain Primary Care Cardiovascular Disease Prevention and Management Guideline [16]. Data were collected across four domains: 1) cardiovascular disease/risk factor screening, 2) drug prescriptions related to CVD, 3) referral to external programs (e.g., referral to smoking cessation program), and 4) clinical test results (e.g., blood pressure readings, lipid profiles, etc.). Process of care data assessed whether recommended care manoeuvres were performed, discussed, or recommended and were recorded as dichotomous indicators, while clinical outcome data were continuous. The Ottawa Hospital Research Ethics Board approved this study (2007292-01H).

### Data analysis

ICC estimates were calculated using an ANOVA approach in which each of the nine geographic regions within the Champlain Local Health Integration Network was treated as fixed strata, corresponding to the study design [17]. ICC estimates were calculated for all patients and separately for patient subgroups defined by condition (i.e., coronary artery disease, diabetes, chronic kidney disease, hypertension, dyslipidemia, and smoking). We compared ICC estimates between practices in which data were collected from a single physician versus those that had multiple participating physicians and between urban versus rural practices. Negative ICC estimates were attributed to sampling error and set to zero [18]. It should be noted that although there are situations that can give rise to true negative ICCs (e.g., when there is competition between clusters), ICCs in the context of cluster randomized trials are generally expected to be positive. ICC estimates may however be negative simply due to chance, particularly when ICC values are close to 0. SAS 9.3 was used for all analyses.

## Results

Table 1 provides a breakdown of the practice and patient profiles. The 84 participating practices were diverse, varying in practice team structure, physician remuneration approach, and rurality (Table 1). Of the 84 practices, 33 were single physician practices, while 51 had multiple physicians on staff (mean: 4, range: 2–19). Of the 51 group practices, 17 only had a single physician consent to participate in IDOCC, and were thus included in the solo physician group for the purposes of this analysis (i.e., 50 solo physicians, 34 multiple physician practices). Patients from such practices had the consenting physician as their main provider; whereas patients from group practices with multiple consenting physicians had different main providers. It is possible that patients in a group practice may be seen by multiple physicians within the same practice, but this likely represents only a small percentage of visits.

**Table 1:** **Baseline characteristics of practices and patients participating in IDOCC**

**Characteristic**	**n (%)**
**Practice Level (N = 84 practices)**	
Use Electronic Medical Records	41 (48.8%)
Practice structure	
Single physician	33 (39.3%)
Multi-Physician Group Practice	51 (60.7%)
Physician remuneration	
Fee for service	45 (52.4%)
Capitation	27 (32.9%)
Salary- Community Health Centres	12 (14.6%)
Practice setting	
Urban practice	70 (83.3%)
Rural practice	14 (16.7%)
**Patient Level (N = 5,140 patients)**	
Age (mean, SD)	66 (12)
Male (n, %)	2,493 (48.6%)
Baseline clinical conditions/risk factors	
Coronary Artery Disease (n,%)	1573 (30.6%)
Peripheral Vascular Disease (n,%)	327 (6.4%)
Stroke/TIA (n, %)	666 (13.0%)
Diabetes (n, %)	2404 (46.8%)
Chronic Kidney Disease (n, %)	975 (19.0%)
Dyslipidemia (n, %)	4289 (83.6%)
Hypertension (n, %)	3959 (77.1%)
Current Smoker (n, %)	1082 (21.1%)

ICC estimates obtained from all 84 primary care practices for process of care and clinical outcomes are presented in Tables 2 and 3 respectively. ICC estimates ranged from 0 to 0.173, with a median of 0.056 (Q1 to Q3, 0.025 to 0.094). The median ICC value for dichotomous process outcomes (0.088) was higher than that for continuous (0.035) clinical outcomes. The largest ICC estimates were for process of care measures looking at waistline measurement (0.173), ACR screening for patients with diabetes (0.167), and two blood pressure readings for patients with chronic kidney disease (0.157).

**Table 2:** **ICC estimates for process of care outcomes**

Quality of Care Indicator*	**N**	**Average cluster size**	**Proportion** ^Ɨ^	**ICC**
**All patients (n = 5140)**				
One blood pressure reading	5133	61	0.93	0.052
Two blood pressure readings	5129	61	0.75	0.111
Lipid profile	5133	61	0.78	0.076
Fasting blood glucose	5133	61	0.81	0.045
eGFR	5133	61	0.80	0.066
ACR	5133	61	0.36	0.122
Smoking status checked	5132	61	0.96	0.088
Waist circumference	5140	61	0.10	0.173
**Patients with Hypertension (n = 3959)**				
One blood pressure reading	3959	47	0.95	0.045
Two blood pressure readings	3957	47	0.80	0.115
Anti-hypertensive medication	3959	47	0.94	0.015
**Patients with Dyslipidemia (n = 4289)**				
Lipid profile	4289	51	0.83	0.050
Lipid lowering medication	4289	51	0.91	0.019
**Patients with Diabetes (n = 2404)**				
HbA1c	2402	29	0.87	0.091
Glycemic Medication	2402	29	0.80	0.041
1 blood pressure reading	2404	29	0.93	0.063
2 blood pressure reading	2404	29	0.76	0.133
Lipid Profile	2404	29	0.81	0.094
Fasting blood glucose	2404	29	0.85	0.097
eGFR	2404	29	0.84	0.087
ACR	2404	29	0.56	0.167
**Patients with Chronic Kidney Disease (n = 975)**				
eGFR	975	12	0.92	0.064
ACR	975	12	0.52	0.123
1 blood pressure reading	975	12	0.95	0.074
2 blood pressure readings	974	12	0.83	0.157
**Patients with Coronary Artery Disease (n = 1573)**				
Medication	1573	19	0.89	0.017
1 blood pressure reading	1573	19	0.92	0.091
2 blood pressure readings	1572	19	0.77	0.137
Lipid profile	1573	19	0.75	0.104
**Patients who smoke (n = 1082)**				
Smoking advice	1082	13	0.53	0.120
Smoking program	1082	13	0.08	0.148
Smoking cessation drug	1082	13	0.23	0.054

*whether specified quality of care indicator was discussed, recommended, or performed during a one year timeframe, ^Ɨ^represents the proportion of patients that received the listed clinical manoeuvre.

eGFR – Estimated Glomerular Filtration Rate; ACR – Albumin-to-Creatinine ratio; HbA1c – Hemoglobin A1c.

**Table 3:** **ICC estimates for clinical outcomes**

**Clinical Outcome**	**N**	**Average cluster size**	**Mean**	**Standard Deviation**	**ICC**
**All patients**					
SBP	4771	57	130.8	16.6	0.054
DBP	4771	57	75.5	10.1	0.094
LDL	3805	45	2.43	0.93	0.035
HDL	3355	40	3.39	0.78	0.020
FBG	4053	48	6.64	2.2	0.023
eGFR	4000	48	72	18.6	0.015
ACR	1589	19	9.4	43.6	0.000*
**Patients with Hypertension**					
SBP	3746	45	132.6	16.6	0.055
DBP	3746	45	76.1	10.2	0.091
**Patients with Dyslipidemia**					
LDL	3395	40	2.39	0.94	0.033
HDL	2997	36	3.38	0.77	0.019
**Patients with Diabetes**					
SBP	2226	27	130.9	16.4	0.038
LDL	1856	22	2.23	0.9	0.054
HDL	1663	20	3.38	0.8	0.039
HbA1c	1999	24	0.074	0.0	0.003
FBG	1986	24	7.83	2.6	0.022
eGFR	1963	23	73.9	19.7	0.016
ACR	1199	14	10.23	44.0	0.000*
**Patients with Chronic Kidney Disease**					
eGFR	859	10	51.5	16.79	0.058
SBP	922	11	130	18.02	0.078
DBP	921	11	72.8	10.73	0.115
**Patients with Coronary Artery Disease**					
SBP	1452	17	128.2	17.75	0.069
DBP	1453	17	72.9	10.12	0.124
LDL	1136	14	2.13	0.78	0.053
HDL	1049	12	3.29	0.75	0.006
**Patients who Smoke**					
SBP	995	12	130.4	17.3	0.013
DBP	994	12	76.7	10.1	0.070
LDL	757	9	2.58	1.0	0.026
HDL	611	7	3.55	0.8	0.018

SBP – Systolic Blood Pressure, DBP – Diastolic Blood Pressure, LDL – Low Density Lipoprotein, HDL – High Density Lipoprotein, FBG – Fasting Blood Glucose, eGFR – Estimated Glomerular Filtration Rate, ACR – Albumin-to-Creatinine ratio, HbA1c – Hemoglobin A1c; *Negative ICCs were considered a result of sampling error and were set to zero.

In general, ICC values were fairly similar across different patient conditions. This may not be surprising as there is substantial overlap between some patient condition subgroups as can be seen from Table 1. For process of care measures (Table 2), medication prescribing across cardiovascular related conditions (i.e., CAD, diabetes, hypertension, and dyslipidemia) tended to have low ICCs (0.01 to 0.04), while ICCs for measuring blood pressure at least twice a year was above 0.1 across all applicable conditions (Table 2). For clinical outcomes (Table 3), all ICC measures were below 0.1, with the exception of measures for diastolic blood pressure for patients with coronary artery disease (0.12) and chronic kidney disease (0.12).

Tables 4 and 5 compare ICC values between practices in which data were collected from a single physician (50 practices) versus multiple physicians (34 practices) for process of care and clinical outcomes, respectively. In general, ICC estimates for process of care measures arising from single physician practices were higher than those arising from multiple-physician practices. ICC values collected from a single physician were on average 3.9 times higher (median: 2.7 times higher) than the value collected from multiple-physician practices, with a maximum difference of 11 times greater for two blood pressure measures per year for patients with diabetes. A similar trend was seen for clinical outcomes as ICC estimates for single physician practices was on average 1.9 times higher (median: 1.8 higher) than multiple physician practices. The largest differences in ICC estimates among all the clinical outcomes were for systolic and diastolic blood pressure measures

**Table 4:** **Comparison of ICC estimates for process-of-care indicators between single versus multiple physician practices**

Quality of Care Indicator*	**ICC**	**ICC**	**Ratio (Single/Multiple)**
	**Single Physician**	**Multiple Physicians**	
	**N = 50**	**N = 34**	
**All patients**			
One blood pressure reading	0.064	0.021	3.06
Two blood pressure readings	0.154	0.029	5.24
Lipid profile	0.109	0.011	10.14
Fasting blood glucose	0.043	0.017	2.56
eGFR	0.066	0.041	1.61
ACR	0.146	0.054	2.70
Smoking status checked	0.080	0.094	0.85
Waist circumference	0.237	0.091	2.59
**Patients with Hypertension**			
One blood pressure reading	0.059	0.015	3.87
Two blood pressure readings	0.160	0.024	6.64
Anti-hypertensive medication	0.019	0.011	1.63
**Patients with Dyslipidemia**			
Lipid profile	0.069	0.009	8.08
Lipid lowering medication	0.016	0.016	1.04
**Patients with Diabetes**			
HbA1c	0.093	0.037	2.51
glycemic medication	0.046	0.028	1.66
1 blood pressure reading	0.080	0.009	8.89
2 blood pressure readings	0.192	0.017	11.42
Lipid Profile	0.109	0.032	3.40
Fasting blood glucose	0.082	0.034	2.40
eGFR	0.080	0.059	1.35
ACR	0.172	0.124	1.38
**Patients with Chronic Kidney Disease**			
eGFR	0.039	0.057	0.69
ACR	0.178	0.033	5.35
1 blood pressure reading	0.089	0.018	5.01
2 blood pressure readings	0.221	0.035	6.37
**Patients with Coronary Artery Disease**			
CAD Medication	0.026	0.009	2.95
1 blood pressure reading	0.108	0.031	3.46
2 blood pressure readings	0.205	0.028	7.35
Lipid profile	0.154	0.018	8.67
**Patients who Smoke**			
Smoking advice	0.072	0.064	1.12
Smoking program	0.136	0.144	0.95
Smoking cessation drug	0.072	0.034	2.11

*whether specified quality of care indicator was discussed, recommended, or performed during a one year timeframe.

eGFR – Estimated Glomerular Filtration Rate; ACR – Albumin-to-Creatinine ratio; HbA1c – Hemoglobin A1c.

**Table 5:** **Comparison of ICC clinical outcomes between single physician versus multiple physicians practices**

**Clinical Outcome**	**ICC**	**ICC**	**Ratio (Single/Multiple)**
	**(Single Physician)**	**(Multiple Physicians)**	
	**N = 50**	**N = 34**	
**All patients**			
SBP	0.082	0.020	4.10
DBP	0.133	0.051	2.60
LDL	0.042	0.029	1.44
HDL	0.024	0.024	1
FBG	0.027	0.020	1.35
egfr	0.019	0.010	1.90
acr	0.000*	0.000*	---
**Patients with Hypertension**			
sbp	0.086	0.020	4.30
dbp	0.130	0.042	3.10
**Patients with Dyslipidemia**			
LDL	0.044	0.023	1.91
HDL	0.019	0.024	0.80
**Patients with Diabetes**			
SBP	0.061	0.015	4.10
DBP	0.108	0.053	2.04
LDL	0.068	0.039	1.74
HDL	0.041	0.047	0.87
HbA1c	0.019	0.000	---
FBG	0.017	0.034	0.50
egfr	0.016	0.018	0.89
**Patients with Chronic Kidney Disease**			
egfr	0.060	0.029	2.07
SBP	0.092	0.055	1.67
DBP	0.131	0.078	1.68
**Patients with Coronary Artery Disease**			
SBP	0.083	0.020	4.15
DBP	0.155	0.084	1.85
LDL	0.048	0.054	0.89
HDL	0.000*	0.038	---
**Patients who Smoke**			
SBP	0.008	0.000	---
DBP	0.114	0.020	5.7

*Negative ICCs were considered a result of sampling error and were set to zero SBP – Systolic Blood Pressure; DBP – Diastolic Blood Pressure; LDL – Low Density Lipoprotein; HDL – High Density Lipoprotein; FBG – Fasting Blood Glucose; eGFR – Estimated Glomerular Filtration Rate; ACR – Albumin-to-Creatinine ratio; HbA1c – Hemoglobin A1c.

Table 6 compares differences in ICC values between urban (N = 70) and rural (N = 14) practices for process of care outcomes. In general, urban practices had higher ICC estimates for process of care indicators, particularly for lipid profile and eGFR measures. In terms of clinical outcomes, ICC estimates were similar between urban and rural practices (results not shown).

**Table 6:** **Comparison of ICC process of care indicators between urban and rural practices**

Quality of Care Indicator*	**ICC: Rural**	**ICC: Urban**	**Ratio**
	**N = 14**	**N = 70**	**(Urban/Rural)**
**All patients**			
One blood pressure reading	0.056	0.052	0.92
Two blood pressure readings	0.119	0.105	0.88
Lipid profile	0.012	0.082	6.91
Fasting blood glucose	0.021	0.047	2.22
egfr	0.016	0.075	4.83
ACR	0.133	0.115	0.86
Smoking status	0.054	0.091	1.69
Waist circumference	0.117	0.167	1.43
**Hypertension**			
One blood pressure reading	0.019	0.050	2.59
Two blood pressure readings	0.085	0.118	1.39
Anti-hypertensive medication	0.017	0.016	0.91
**Dyslipidemia**			
Lipid profile	0.014	0.055	4.08
Lipid lowering medication	0.027	0.020	0.74
**Diabetes**			
HbA1c	0.026	0.090	3.48
glycemic medication	0.027	0.045	1.68
1 blood pressure reading	0.067	0.065	0.97
2 blood pressure reading	0.166	0.123	0.74
Lipid Profile	0.014	0.105	7.68
Fasting blood glucose	0.057	0.097	1.69
egfr	0.021	0.097	4.56
ACR	0.142	0.164	1.16
**Chronic Kidney Disease**			
egfr	0.005	0.072	14.61
ACR	0.112	0.129	1.16
1 blood pressure reading	0.012	0.088	7.10
2 blood pressure reading	0.063	0.177	2.82
**Coronary Artery Disease**			
CAD Medication	0.029	0.016	0.53
1 blood pressure reading	0.041	0.093	2.23
2 blood pressure reading	0.053	0.147	2.77
Lipid profile	0.006	0.103	15.88
**Smoking**			
Smoking advice	0.118	0.113	0.96
Smoking program	0.038	0.164	4.32
Smoking cessation drug	0.090	0.050	0.56

*whether specified quality of care indicator was discussed, recommended, or performed during a one year timeframe.

eGFR – Estimated Glomerular Filtration Rate; ACR – Albumin-to-Creatinine ratio.

### Sample calculation

We now present a hypothetical example to illustrate the potential impact of using inaccurate ICC estimates on the design of a cluster randomized trial. Consider a planned cluster randomized trial aimed at improving the delivery of evidence-based care for high risk patients with hypertension. The primary outcome for the trial is patient systolic blood pressure. The target population consists of solo physician primary care practices. The study is being planned to detect a 5 unit mean difference in systolic blood pressure between intervention and control practices using a two-sided test at the 5% level of significance with 90% power, assuming an average cluster size of 20 patients per practice. If the estimated ICC and SD for multiple physician practices were used from Table 5 (ICC = 0.020, SD = 16.5, VIF = 1.4), the target number of clusters would be 16 practices per arm. On the other hand, if the higher ICC estimate for solo physicians had been used (Table 5: ICC = 0.086, SD =16.6, VIF = 2.6), the sample size requirement would be 31 practices per arm.

## Discussion

To the best of our knowledge, this is the first article to present ICC values for a range of cardiovascular-related outcomes collected from primary care practices in Canada. The ICC values derived from the IDOCC study are in the range of previously reported estimates that have been obtained from primary care settings [1,14,19-21]. In general, ICC values for process of care variables were higher than those for clinical outcomes, which is consistent with findings from other studies [10,22]. Intuitively, this is not surprising as clinical outcomes within a given cluster have a greater potential for variability, as each patient will have different levels of compliance and responses to a given treatment.

ICC estimates related to medication prescriptions/recommendations tended to have smaller values than ICCs for other process of care indicators, reflecting little variability amongst practices in prescribing medications for high risk patients. As has been shown in other primary care studies, there is clear evidence and physician agreement on the importance of prescribing medications to high risk patients with cardiovascular disease, diabetes, hypertension and/or dyslipidemia [23-26]. On the other hand, the largest ICC values amongst the process of care indicators were for waistline measurement, two blood pressure screenings per year, and ACR measurement, reflecting relatively high between practice variability for these measures. Unlike medication prescribing for high risk patients, there is likely less agreement amongst physicians regarding the need and/or appropriateness of doing these process manoeuvres. For example, although the importance of waistline measurement has been widely publicized in predicting all-cause and cardiovascular-related mortality, [27] previous studies have demonstrated that some physicians do not do this screening test due to a lack of time, extra workload and financial implications, while others feel uncomfortable measuring waists or are concerned that patients might get embarrassed [28].

Overall, ICC levels for clinical outcomes were relatively low (<0.1) with the exception of values for diastolic blood pressure. This finding is in line with findings presented by Parker et al. [19], which found diastolic blood pressure measurements to have the largest ICC value from a group of clinical outcome markers collected from primary care practices in Rhode Island and Southeastern Massachusetts. The high ICC value likely reflects the hypertension management style of individual physicians [19].

The differences in ICC values between single versus multiple physician practices and those between urban versus rural practices highlight the importance of understanding the context in which ICC estimates are derived before using them in sample size calculations. It can be challenging to find an ICC estimate that was derived from a population and setting that matches the planned study. Researchers may have no choice but to use whatever ICC estimates are available for the outcome of interest, with the assumption that differences in context may only moderately impact sample size estimates. Using an ICC estimate that is too small can result in a substantially underestimated sample size and prevent drawing any definitive conclusions about the results of an intervention. Therefore, it is important to closely examine published ICC estimates to determine whether they are relevant to the planned trial. At the same time, when reporting an ICC estimate, researchers should clearly describe characteristics of the practices included in the trial. Our estimates pertain to the primary care setting; Campbell et al. [10] found that ICCs were significantly higher for secondary care outcomes compared with primary care outcomes.

There were several limitations in this study. Since practices in this analysis consented to take part in the IDOCC study, there is a potential selection bias. Practices that opted to participate in IDOCC are likely more highly motivated and higher performing than provincial averages. As such, it is possible that participating practices have inherent similarities that could decrease between practice variance and result in ICC values that are too small. However, considering that many primary care studies are voluntary, the estimates presented in this paper are likely representative.

In addition, the data for this study were collected from practices across Eastern Ontario only. As such, the results may not be generalizable to primary care studies conducted in jurisdictions with healthcare systems that are very different than in Ontario. However, ICC estimates found in this study were in line with values presented in primary care studies conducted in different countries [14,19].

We have used a simple one-way ANOVA to calculate ICC estimates. This method is commonly used to calculate ICCs for any type of outcome but the resulting value must be interpreted with care if the assumptions of analysis of variance are violated. Eldridge et al. [29] review several alternative definitions of ICCs in cluster randomized trials. Finally, large studies are required to estimate ICCs with a reasonable degree of accuracy. Although our main estimates are based on a relatively large sample size, we have not provided confidence intervals around our estimates. Readers using these ICCs for sample size calculation may therefore need to consider the degree of uncertainty associated with these estimates; in particular, the number of rural practices (N = 14) examined in this study was relatively small. Ukoumunne [30] and Zou and Donner [31] review confidence interval methods for ICCs in CRTs.

## Conclusions

To the best of our knowledge, this article presents the most comprehensive summary of ICC values related to cardiovascular-related outcomes collected from Canadian primary care practices. The ICC estimates presented in this study cover a wide range of conditions and risk factors that can be used to aid in the design of future cluster randomized trials in primary care settings. Furthermore, we observed substantial differences in ICC estimates obtained from single physicians versus a group of physicians; this demonstrates the importance of understanding the context in which ICC values are determined before using them in sample size calculations. Failure to take these differences into account can have substantial implications for the design of a cluster randomized trial.

## Abbreviations

ACR: Albumin-to-creatinine ratio
CAD: Coronary artery disease
CVD: Cardiovascular disease
eGFR: Estimated glomerular filtration rate
ICC: Intracluster correlation
IDOCC: Improved Delivery of Cardiovascular Care
SD: Standard deviation
TIA: Transient Ischemic Attack
VIF: Variance inflation factor
